# Thermal Remediation of Soil Contaminated with Polycyclic Aromatic Hydrocarbons: Pollutant Removal Process and Influence on Soil Functionality

**DOI:** 10.3390/toxics10080474

**Published:** 2022-08-16

**Authors:** Chenfeng Liu, Huading Shi, Chen Wang, Yang Fei, Ziyu Han

**Affiliations:** Technical Centre for Soil, Agriculture and Rural Ecology and Environment, Ministry of Ecology and Environment, Beijing 100012, China

**Keywords:** desorption, basic soil properties, thermal kinetics, polycyclic aromatic hydrocarbons, benzo(a)pyrene, removal efficiency, soil functionality

## Abstract

Thermal remediation has been widely used for the removal of polycyclic aromatic hydrocarbon (PAH) from contaminated soil. The method has a high removal rate for semi-volatile organic pollutants; however, soil functionality is affected by the method because of the alteration of the soil properties. In this study, experimental soil was impregnated with phenanthrene (Phe), pyrene (Pyr), and benzo(a)pyrene (BaP); after natural air-dry aging, the thermal remediation experiment was carried out, using a tube-furnace and thermal gravimetry–Fourier transform infrared (TG-FTIR) equipment. More than 84% of the Phe and Pyr were lost in the aging stage, whereas the BaP was stable with 41% retention in the soil. After the thermal treatment, the desorption and decomposition of the pollutants and organic matter led to the removal of the PAHs; about 1% of the PAHs remained in the soil treated at 400 °C. The presence of the PAHs can promote the thermal reaction by slightly reducing the reaction activation energy by ~7−16%. The thermal remediation had a significant influence on the physical properties of the soil and destroyed the bioavailability by reducing the organic matter content. Therefore, a comprehensive consideration of effective PAH removal while preserving soil functionality may require a low temperature (100 °C) method for thermal remediation.

## 1. Introduction

The release of polycyclic aromatic hydrocarbons (PAHs) through human activity, especially industrial manufacturing, has created great environmental concerns due to the high toxicity and persistence of these species. Generally, the sources of PAH pollution in soil are extensive, including combustion of fossil fuel [1], automobile exhaust [2], waste treatment [3], etc., while the methods of soil treatment are limited. Bioremediation [4], soil washing (solvent extraction) [5], phytoremediation, photocatalytic degradation, electrokinetic remediation [6], and chemical oxidation [7] are the most common methods. Besides, the thermal remediation of PAH-polluted soil is also regarded as a potential method with high efficiency [7,8].

For instance, China has been regarded as the ‘world factory’ for a long period of time; with the development of urbanization, a large number of factories must be relocated. It is not uncommon that the polluted sites which are contaminated by semi-volatile organic compounds, such as PAHs and petroleum hydrocarbons (PHCs), are located in densely populated areas. Normally, thermal remediation is a viable option for specific urban areas where the thermal remediation cost is justified by the high land commercial value and the revenues from site redevelopment.

Thermal remediation (or thermal desorption of soil pollutants) refers to a physical method of separating the pollutants from the soil through phase transformation [9]. Generally, the pollutants treated through thermal remediation include PAHs [7], semi-volatile polychlorinated biphenyls (PCBs) [9], petroleum hydrocarbons [10], and other semi-volatile organic contaminants. The current reports have documented that thermal remediation can be applied to both farmland [11] and in situ soil. The temperature is an important factor in this method, where some pieces of research have indicated that only about a 10% PAH removal from polluted soil can be achieved at a low temperature (60–150 °C) [12]. A study reported that 99% of 16 US EPA priority PAHs can be removed from contaminated soil after about an 8 h 400 °C thermal remediation, while the soil’s basic properties would be changed significantly [13].

Actually, there is no universally appropriate thermal treatment technology. Rather, the appropriate choice depends on the contamination and on site-specific considerations, such as the soil properties [14]. The soil particle size, soil stability, and compressive strength increased after the thermal remediation, based on the analysis between the soil parameters before and after treatment. The soluble salt content increased after the thermal remediation, indicating an increase in the soil salinization. Nevertheless, due to their complex chemical structure, most of the PAHs will be desorbed or decomposed at a higher temperature [15]. However, the thermal process has a high impact on other soil functionality [16] by affecting the soil organic matter, soil texture and mineralogy, soil pH, plant-available nutrients and heavy metals, soil biological communities, and the ability of the soil to sustain vegetation [17]. The changes in the soil properties are a concern for re-utilization. At a high temperature, the structure of the organic matter and some mineral substances in the soil will be destroyed [13], whereas at a low temperature, the effective removal of pollutants will be limited.

The thermal remediation of the soil can rapidly reduce the PAHs’ contamination depending on the process temperature, but the basic properties of the soil will be altered. The purpose of this study is to discuss the effect of thermal treatment on altering the functionality (physical and chemical properties and bioavailability) of PAH-contaminated soil. The results of this research can supply information for managers to balance the requirements for the reduction in contamination and deterioration of soil functionality.

## 2. Materials and Methods

### 2.1. Materials

The experimental soil was collected from the northwest of China (Gansu Province), which was loess soil; the basic properties of the soil that may be related to soil function are presented in Table 1. After drying and removing the impurities, the soil for the experiment maintained its original state. The soil for detection was ground into small particles and passed through the aperture of a 74 μm sieve.

Focused ring structure PAHs phenanthrene (Phe), pyrene (Pyr), and benzo(a)pyrene (BaP) were used as the simulated soil contaminants; the basic properties of the PAHs are presented in Table 2. Each reagent (1 mL 125 mg·L^−1^) was dissolved in 100 mL of 5% acetonitrile solution, and impregnated into 100 g of experimental soil, followed by natural drying. The theoretical concentration of the pollutant in the simulated contaminated soil was 1.25 mg·kg^−1^. Then, the contaminated soil was aged in a cool, dry place for two weeks before thermal treatment.

The experimental group of this study is shown in Table 3.

### 2.2. Experiment

**Figure 1:**
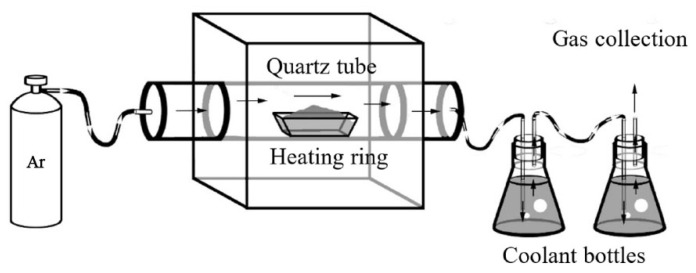
Schematic of lab-scale soil heating apparatus.

A quartz (Figure 1) tube furnace was used to heat the soils; the length of the quartz tube in the tube furnace was 1 m, and the inner diameter was 80 mm. The soil sample (5 g) was placed in a porcelain boat then pushed into the heating zone, and the quartz tube was heated to respective temperatures of 50, 100, 200, 400, and 800 °C at a rate of 10 °C·min^−1^. The reactor was continuously purged with high-purity argon at 0.5 L min^−1^ to sweep the released gases from the quartz tube to imitate the extraction conditions of underground soil without oxygen. The soil was retained at the target temperature for 2 h. Argon purging was used during the whole experiment, including the cooling stage. The products of the extraction were collected in cooling bottles with the absorption liquid. Five parallel samples per treatment were collected for detection.

At low temperatures (60−150 °C), the rate of PAH removal from the soil only reached ~7%, even after treatment in the oven for 1 week [14]. At a higher temperature (>200 °C), more than 81.4% of the organic compounds were removed in 20 min [18]. Therefore, in this study, the treatment time was maintained at 30 min to investigate the influence of temperature on PAH removal.

The weight loss of the soil and the exhaust composition were detected by thermal gravimetry–Fourier transform infrared (TG-FTIR) analysis. The experiment was carried out on a thermal gravimetric analyzer (TGA, SDT-Q600, New Castle, DE, USA), where the 20 mg samples were placed in the burning room and pyrolyzed by increasing the temperature from room temperature to 800 °C at a heating rate of 10 °C·min^−1^. A high-purity argon stream was continuously passed into the equipment. The exit gas was detected by combined Fourier transform infrared spectroscopy (FTIR, Vertex70, Bruker, Germany).

### 2.3. Soil Properties Analyses

The soil samples were taken to the laboratory, air-dried, and sieved (<2 mm) for the laboratory analyses of the pH and cation exchange capacity (CEC). The soil organic matter was tested by colorimetry, following dichromate oxidation by boiling with a mixture of potassium dichromate and sulfuric acid [19]. The total N was measured using the Kjeldahl acid-digestion method [20]; the total K and P were determined after digestion of the sample with NaOH solution; the available P was extracted with NaHCO_3_; the available K was determined by dissolving the sample with HNO_3_, and all of the solutions were analyzed by flame spectrophotometry. The urease activity was determined via colorimetry by exploiting the color development of the sodium phenate and sodium hypochlorite. The soil particle size and specific surface area were analyzed with a laser particle analyzer. For the accuracy of the data, each soil sample which includes the parallel sample was divided into three portions for detection. The detailed information of the experiments can be found in the Supplementary Materials.

### 2.4. Data Analysis

Soil is a complex compound. Theoretically, the biomass can be considered as a single component substance, therefore first-order thermal decomposition kinetics can be used to describe the pyrolysis process [21]. Generally, a mathematical method is used for calculating the activation energy, based on the TGA experiments. The equation describes the temperature dependence of the reaction rate:

Dα/dt = kƒ(α); α = (m_0_ − m)/(m_0_ − m_∞_) × 100% (1)

where α is the degree of decomposition; m_0_, m, m_∞_, and ƒ(α) in the reaction model, respectively, represent the initial, certain time, and final residual mass of the sample after pyrolysis and a functional dependent on the mechanism; t is the time; and k is the Arrhenius rate constant, which can be described as:

k = Aexp(−E/RT); T = tβ(2)

where A is the frequency factor; E is the reaction energy; R is the gas constant (8.314 J mol^−1^ K^−1^); T is the absolute temperature; and β is the heating rate. The functional form ƒ(α) depends on the reaction mechanism. By combining Equations (1) and (2), the function can be represented as:

dα/dt = Aexp(−E/RT) ƒ(α)(3)

For the TGA process, the heating rate can be regarded as: β = dT/dt. By combining with Equation (3):

dα/dt = (A/β)exp(−E/RT) ƒ(α) (4)

The deformation formula is as follows:

dα/ƒ(α) = (A/β)exp(−E/RT)dT (5)

The integral is represented as:

ln(−ln(1 − α)/T2) = ln(AR/βE) − E/RT(6)

If the coefficient of association from the plot of y = ln(−ln(1 − α)/T2) versus x = 1/T is high, the formula can describe the pyrolysis process well. The slope of the plot is −E/R and the intercept is ln(AR/βE).

## 3. Results and Discussion

### 3.1. Efficiency of PAH Removal from Soil by Thermal Remediation

**Figure 2:**
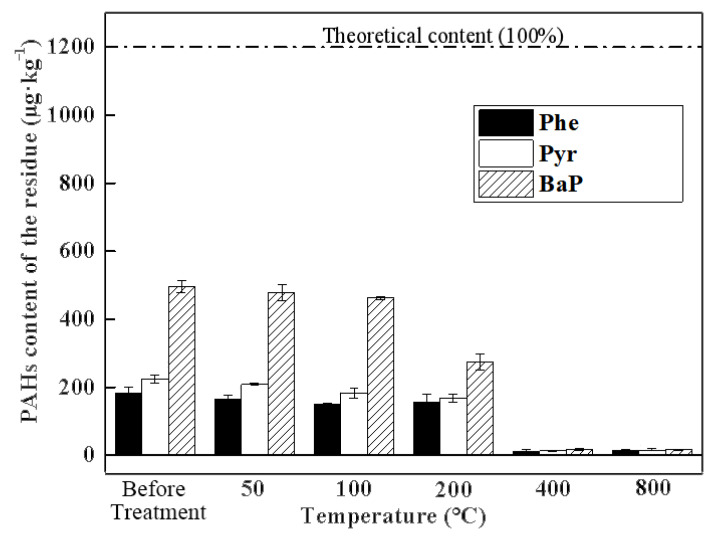
Efficiency of PAH removal from soil using tube furnace.

The efficiency of the PAH removal from the soil in the tube furnace is presented in Figure 2, where the remaining amounts of Phe, Pyr, and BaP in the soil were 181.26 ± 18.26 μg·kg^−1^ (15%), 223.52 ± 11.31 μg·kg^−1^ (18%), and 496.51 ± 18.27 μg·kg^−1^ (41%), respectively, which are reduced compared to the original value for the theoretical simulated contaminated soil of 1250 μg·kg^−1^ (100%). The differences between the initial values and the theoretical value may due to the soil pre-treatment process, where the PAHs diffused into the soil particles and were volatized with solution during the air drying process [22]. The molecular size and volatility were the key factors in this stage; therefore more BaP, which is a more stable compound, was retained.

During the thermal treatment, the temperature significantly influenced the PAH removal efficiency. Before 100 °C, the content of the PAHs decreased slightly. When the temperature was raised to 200 °C, the content of BaP declined from 38% to 23%, whereas that of Phe and Pyr decreased by about 1%. When the temperature was increased to 400 °C, the residual amount of all three of the PAHs decreased to 0.5~1%, and there was no significant difference at 800 °C.

However, the PAHs were removed in the following order: Phe, Pyr, and BaP, depending on the specific characteristics (molecular structure, boiling point, and polarity), where the order is dependent on the number of rings and corresponds to the chromatographic elution order.

The loss of PAHs during the soil preparation step may due to the air drying process. Phe and Pyr are semi-volatile pollutants [23], which can be desorbed from soil in the low-temperature phase (<200 °C) during thermal remediation. In this study, the results showed the limited removal of the three PAHs (below 5%) under the 200 °C treatment, which may because most of the semi-volatile pollutants had been desorbed already, since the aging process. Moreover, Phe and Pyr have small molecular sizes, which leads to a faster aging rate. Therefore, the actual content of Phe and Pyr was lower than that of BaP. The desorption phenomenon is related to the breaking of the bonds between the soil particles and organic compounds. With the increasing temperature, the saturated vapor pressure increased [24], resulting in more effective desorption of the organic compounds. The chemical reactions of the three PAHs, with the other compounds volatilized from soil, occurred at a high temperature (>400 °C), which has environmental health consequences such as the apparently augmented volatilization of hazardous substances in soil, such as PAHs, petroleum hydrocarbon, or diesel [17]. During the thermal process, the macromolecular PAHs may be recombined through some mechanism to form intermediate species [3], which was observed in this study where the content of BaP at 800 °C was slightly higher than that at 400 °C.

Overall, the reduction in the PAH content of soil in this study can be divided into four steps, as indicated in Table 4.

### 3.2. Thermal Kinetics of PAH-Contaminated Soil

**Figure 3:**
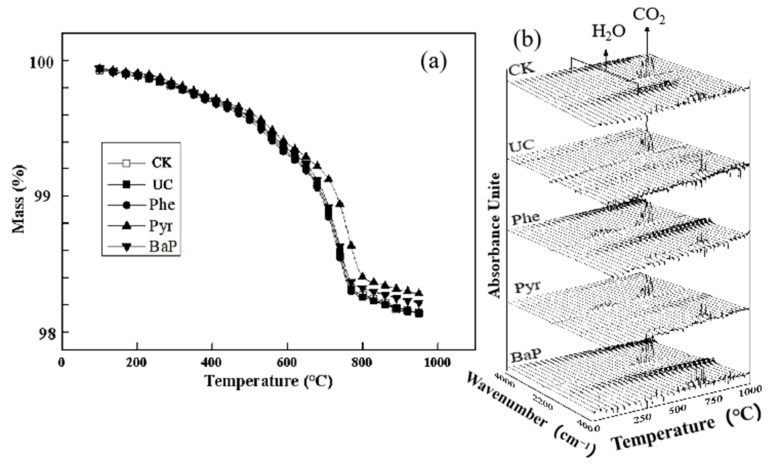
Thermogravimetric curve (**a**) and TG-FTIR (**b**) spectra of contaminated soil.

The dynamic soil changes during heating under anoxic conditions were evaluated using a TG-FTIR analyzer. The thermogravimetric data for the soil are presented in Figure 3a, where the weight change of all of the five groups of sois samples was limited to 2% from room temperature to 900 °C, and more than 1% weight loss occurred at 600−800 °C. The soil impregnated with PAHs lost more weight during heating.

Using the data from the Thermogravimetric (TG) curve, the activation energy E for the reaction from beginning to 400 °C can be calculated from the fitting result, as shown in Table 5. The results suggest that the activation energy of Phe, Pyr, and BaP were lower than that of CK and UC, which indicated that impregnation of Phe, Pyr, and BaP into the soil particles can promote the thermal process by reducing the energy consumption of the soil, possibly due to the azeotropy of the PAHs with the primary minerals [25], and also promotes the pyrolysis of the pollutants, due to organic matter such as cellulose and lignin [21].

During the thermal remediation of the contaminated soil, thermosynthesis and thermolysis occur along with the pyrolysis reactions. The results of the TG-FTIR experiments are presented in Figure 3b, which suggest that during the pollutant decomposition, the majority of the components of the gas that discharged from the contaminated soil were similar to those of the original soil, for example throughout all of the process for H_2_O and mainly from 500 °C for CO_2_.

### 3.3. Changes in Soil Properties after Thermal Remediation

#### 3.3.1. Organic Matter

**Figure 4:**
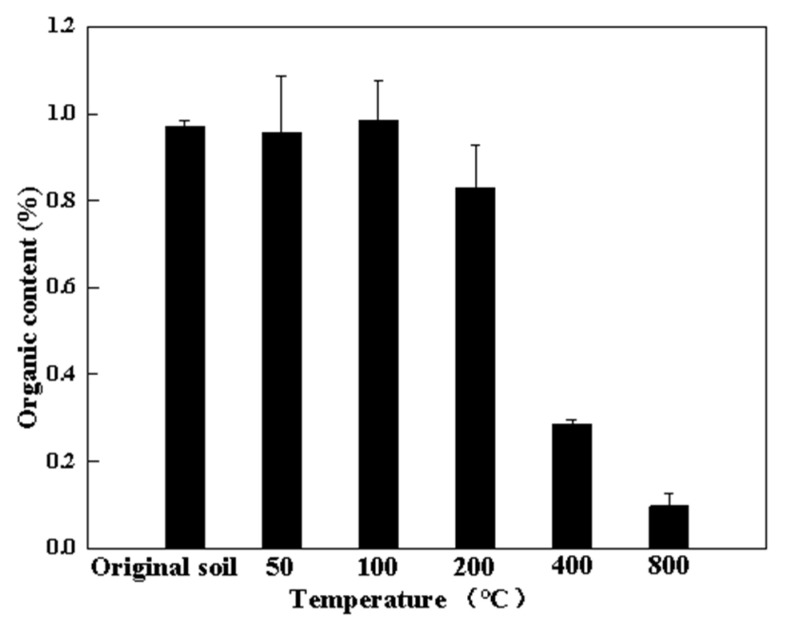
Content of organic matter in soil during thermal remediation.

The changes in the organic matter of soil samples of the UC group through thermal treatment are shown in Figure 4, which indicates that before 200 °C, there was no obvious change in the organic matter of the soil, but when the temperature was raised to 200 °C, the organic matter content decreased from 1.1% to 0.8%. When the temperature reached 400 °C and 800 °C, the organic matter content declined to 0.28% and 0.16%, respectively.

When the temperature was raised to 100 °C, the evaporation and pyrolyzation of the organic matter in the soil were reinforced, leading to reduced organic matter [26]. Generally, in the solid-water-air equilibrium systems, the hydrophobic compounds are positively correlated with soil organic matter [27], which may be a reason for the loss of the organic matter in this study. However, thermal desorption caused significant changes in the composition of the soil organic matter, which is the indirect reason for the change in the physical properties and bioavailability.

#### 3.3.2. Physical Properties

**Figure 5:**
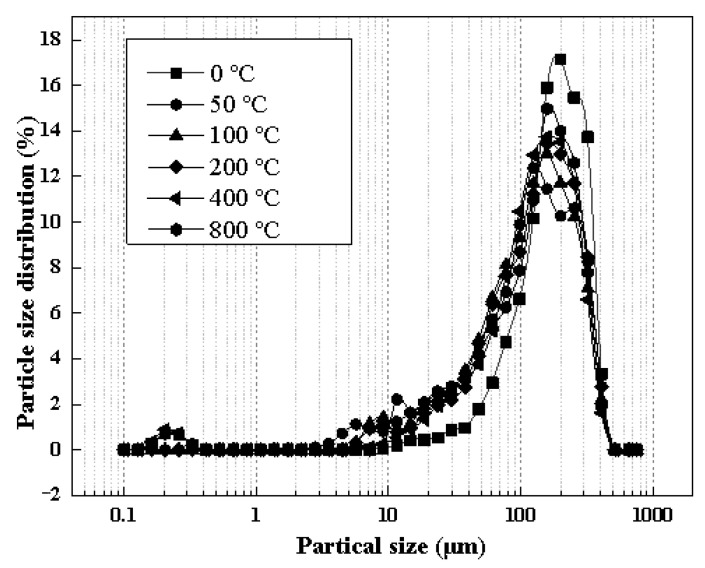
Influence of temperature on particle size of soil.

The influence of the temperature on the particle size of the soil is shown in Figure 5, which indicated that the soil particle size was concentrated in the 10−140 μm range. However, compared with the original soil (UC group), after thermal decomposition, the soil particle size decreased.

The soil particle size has a critical impact on the amount, form, and characteristics of the adsorbed pollutants [28,29]. The ability of soil to adsorb organic pollutants is affected by the particle size of the soil; generally, soil with a smaller particle size had a stronger adsorption capacity for the organic pollutants. Moreover, the pollutant content, source, components, and soil properties can also affect the behavior of PAHs during the pyrolysis process. As research indicated, at the same temperature, the smaller soil particles led to a higher removal and greater decomposition of the organic compounds [9]. The result might be ascribed to easier heating and fewer transfer limitations of the fine particles than the coarse particles, furthermore, it is quite common that for an effective thermal treatment of clayey soils, higher temperatures are required than for coarser soil [17]. Therefore, decreasing the soil particle size could significantly impact the thermal desorption of the PAHs from the soil.

The pH of the soil heated at 50 °C was lower than that of the original soil, attributed to the release of the organic acid. With an increasing treatment temperature, the pH of the soil increased slightly, due to the volatilization of the acids. Therefore, the pH change following soil remediation may be caused by the decomposition of organic acids and the release of alkali cations during the oxidation of the organic matter [10].

The reduction of the organic matter led to a decrease in the amount of the negatively charged organic colloid, which is the main reason for the reduced CEC content. The CEC is also affected by the change in the pH, as the pH has a significant impact on the charge. Generally, the negatively charged species increase at a higher pH [17]. Although in this study, the variation of the pH was within a narrow range, from 7.4 to 8.5, the pH still affected the CEC due to charge variations.

#### 3.3.3. Bioavailability

The remediation via the thermal approach removes the pollutants from the soil. However, it also affects the bioavailability of the soil in terms of fertility and microorganisms. To investigate the possible effects of thermal treatment on soil bioavailability, this research assayed the N, P, K, and an enzyme as the key indicators of the bioavailability.

**Figure 6:**
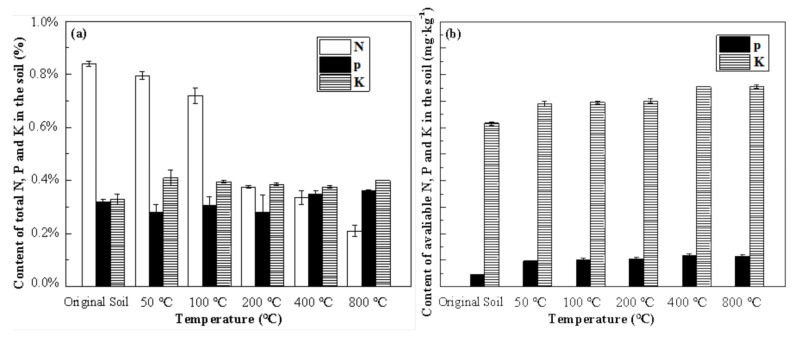
Influence of temperature on soil fertility: (**a**) total N, P, and K; (**b**) available P and K.

Heating the soil produced slight increases in the total potassium, and the increases were enhanced by a decrease in the clay colloid [16]. As the temperature increased in the 100−200 °C range, the total N decreased significantly, which correlated with the observed decrease in the organic matter content, which may be due to the desorption and pyrolyzation of the organic matter (Figure 6). The total P and K of the soil fluctuated slightly, while the available P and K increased, due to the release of the adsorbed P and K into the soluble state at a higher temperature, and the decomposition of the organic matter may also release available P and K as soluble ash [30]. Moreover, organic phosphorous can be converted to inorganic phosphorous, increasing the available P_2_O_5_ [31]. Increasing the temperature led to a rise in the concentration of the available P and K, which may have beneficial effects on plant growth, whilst the total N followed the opposite trend.

**Figure 7:**
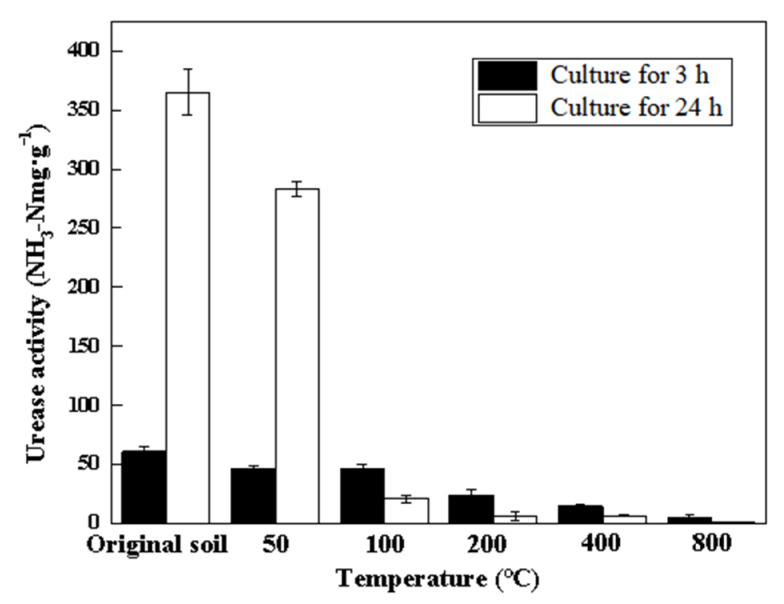
Influence of temperature on soil enzyme sensitivity.

The urease activity after culturing for 3 and 24 h for each temperature stage is shown in Figure 7. Before 50 °C, the urease activity of the 24-h culture was much higher than that of the 3-h culture, which indicated that the microbial population for enzyme production was adequate [32]. The urease activity decreased significantly when the temperature reached 100 °C for the 24-h culture. However, for the 3-h culture this happened when the temperature reach to 200 °C. This result might be caused by protein denaturation and the induced death of the microbes.

The enzymes were sensitive to thermal treatment, which is in agreement with the observed behavior of phosphatase and invertase in another study [33]. The results suggest that the microorganism population was severely destroyed by the high temperatures. From this point of view, thermal remediation at temperatures lower than 100 °C is the environmentally friendly approach for contaminated soil.

## 4. Conclusions

The changes in the PAH concentration and various properties related to soil health during thermal remediation were evaluated. The reduction in the PAH content in the contaminated soil could be divided into aging, desorption, decomposition, and ashing stages. The BaP pollutant was more stable than Phe and Pyr in the aging process; therefore, the majority of BaP would be released during the thermal decomposition stage. The heating process was promoted by the PAHs by reducing the energy required for the reactions. A high temperature (>400 °C) had a significant influence on the physical properties of the soil and degraded the bioavailability by reducing the organic matter. Therefore, a comprehensive consideration of the PAH removal and preservation of soil functionality suggests that a low temperature (<100 °C) treatment would be reasonable for thermal remediation, in case the removal of pollutants is adequate to meet the remedial objectives.

## Figures and Tables

**Table 1:** Properties of the original loess soil from Gansu province of China.

Properties	Organic Matter (%)	pH	CEC (cmol·kg^−1^)	Urease Activity (NH3-Nmg·g^−1^)		Nutrient (%)			Particle Size (μm)			Specific Surface Area (m^3^·kg^−1^)
				3 h	24 h	N	P	K	D90	D75	D50	
Soil	0.93	7.7	3.05	61.25	364.89	0.0084	0.0032	0.0033	312.42	242.411	168.247	496.43

**Table 2:** Selected PAHs and their basic physical properties.

Compound	Molecular Weight (g·mol^−1^)	Boiling Point (°C)	Heat of Vaporization (kJ·mol^−1^)	Chemical Structure
Phenanthrene (Phe)	178	340	52.7	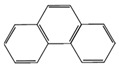
Pyrene (Pyr)	202	404	65.8	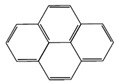
Benzo(a)pyrene (BaP)	252	495	71.1	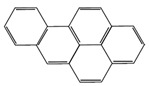

**Table 3:** Experimental group of the thermal remediation treatment.

Experimental Group	CK	UC	Phe	Pyr	BaP
Treatment	Original soil after drying and removing impurities	Impregnated by 5% acetonitrile solution, and aged in cool dry place for two weeks before thermal treatment.	Impregnated by Phe solution and aged	Impregnated by Pyre solution and aged	Impregnated by BaP solution and aged

**Table 4:** Four steps of thermal remediation.

Stage	Duration	Phenomenon and Mechanism
Aging	Until heating	The PAH content was much lower than the original value, especially for Phe and Pyr due to the semi-volatile properties and smaller molecular size.
Desorption	Under 200 °C	The content of Phe and Pyr decreased slightly, while more than 15% of the BaP lost in this stage can be regarded as continuing of aging of persistent pollutant.
Decomposition	200 °C to 400 °C	Boiling point of the PAHs was approximately 400 °C, and the majority of the organic matter was decomposed into low molecular weight matter, which can promote volatilization of the pollutants. Therefore, the main loss of the heating process occurred in this stage.
Ashing	Over 400 °C	The color of the soil become gray due to ashing of the organic carbon, whereas the PAH content did not obviously change.

**Table 5:** Kinetic parameters for the PAH-contaminated soil.

Soil	Correlation Coefficient	Activation Energy (kJ·mol^−1^)
CK	98.56	1337.02
UC	98.33	1324.12
Phe	98.78	1217.33
Pyr	96.12	1101.24
BaP	99.54	1231.58

## Data Availability

All data included in this study are available upon request by contact with the corresponding author.

## Supplementary Materials

The following supporting information can be downloaded at: https://www.mdpi.com/article/10.3390/toxics10080474/s1, Section S1: Detection Methods; Section S2: Supplementary data.
