# Supplementary material for: Thermal Remediation of Soil Contaminated with Polycyclic Aromatic Hydrocarbons: Pollutant Removal Process and Influence on Soil Functionality
Source: Toxics. 2022 Aug 16;10(8):474. doi: 10.3390/toxics10080474 (PMC9416386; doi:10.3390/toxics10080474)
Supplement: Supplementary file 1 [file toxics-10-00474-s001.zip › toxics-1858116-supplementary.pdf]

toxics

MDPI

Supplementary Materials: Thermal Remediation of Soil Contaminated with Polycyclic Aromatic Hydrocarbons: Pollutant Removal Process and Influence on Soil Functionality

Chenfeng Liu, Huading Shi, Chen Wang, Yang Fei and Ziyu Han *

1. Detection Methods

Soil pH: 10 g through 1 mm meshes soil was prepared in 25 mL beaker, 10 mL 0.01 mol·L^{-1}$CaCl_2$ solution was added and mixed fully with soil. After standing 30 min, a pH meter (PHSJ-4A, Shanghai Leici, China) was used for pH value detection.

Soil CEC: 2 g through 1 mm meshes, soil was prepared in 25 mL plastic bottle, 20 mL ammonium oxalate-ammonium chloride solution as added, then lid covered and oscillated on thermostatic oscillator for 15 min at rate of 170 r·min^{-1}, then 10 mL sample was prepared in Kjeldahl bottle, settled on azotometer (SR-04D, Sheyanyiqi, China) and recorded the volume of hydrochloric acid.

Soil PAHs: The PAH content of the soil was measured using an ultrasonic method and detected by a gas chromatograph-mass spectrometer (7000B, Agilent Technologies, Santa Clara, CA, USA). The extraction solution was n-hexane and tetrachloromethane, and the detection temperature was 280 ℃.

2. Supplementary data

Results of changing of pH and CEC though the thermal treatment were showed as following table.

Table S1. Changing of pH and CEC though the thermal treatment.

Soil	pH	CEC（**cmol·kg^{-1}**）
Original soil	7.7	3.05
50℃	7.4	2.91
100℃	7.5	3.02
200℃	8.0	3.02
400℃	8.4	3.05
800℃	8.5	3.05
